# Shell-Forming Stimulus-Active Hydrogel Composite Membranes: Concept and Modeling

**DOI:** 10.3390/mi11060541

**Published:** 2020-05-26

**Authors:** Adrian Ehrenhofer, Thomas Wallmersperger

**Affiliations:** Institute of Solid Mechanics, Technische Universität Dresden, 01062 Dresden, Germany; thomas.wallmersperger@tu-dresden.de

**Keywords:** hydrogels, rollable/flexible displays, modeling and simulation, shell-forming, corrugated sheets

## Abstract

The swelling of active hydrogels combined with passive layers allows the design of shell-forming structures. A shell-like structure offers different properties than a flat structure, e.g., variations in bending stiffness across different directions. A drastic increase of the bending stiffness is favorable e.g., in rollable/flexible displays: in their unrolled form, they have to be stiff enough to resist bending due to dead weight. At the same time, they have to be flexible enough to be rolled-up. This can be achieved by shell-forming. In the current modeling and simulation work, we present a basic concept of combined active–passive composites and demonstrate how they form shells. As the example material class, we use hydrogels with isotropic swelling capabilities. We demonstrate how to model the combined mechanical behavior with the Temperature-Expansion-Model. Afterwards, we show numerical results obtained by Finite Element simulations. We conclude that the envisioned structure has a great potential for obtaining soft rollable sheets that can be stiffened by intrinsic activation.

## 1. Introduction to Hydrogel Composite Membranes and Shells

Stimulus-active hydrogels can be combined with passive layers to form active composite membranes [1]. Thus, the isotropic swelling capabilities that characterize hydrogels [2] can be used in combination with the high stiffness of the passive material. In our previous works, we investigated a setup composed of the temperature-sensitive hydrogel poly(N-isopropyl acrylamide) (PNiPAAm), which was combined with polyethylene terephtalate (PET). The system shows high potential in the application as switchable membrane for the handling of blood samples [1,3,4].

In the current work, we demonstrate the potential of active–passive composites for structures with switchable stiffness to stabilize rollable displays. Rollable/flexible displays are a current trend in portable devices [5,6,7]. In their actual form, they are mostly combined with hard casings for stabilization. Other approaches use e.g., bistability mechanisms [8].

Hydrogels are polymers in watery environment with the capabilities of swelling and deswelling due to external stimuli [2,9]. In the micro-mechanical context, hydrogels with swelling capacities can be used e.g., for flow control [10,11], sensor setups [12,13] or micro-actuators [14,15]. Experimental data for the mechanical behavior of hydrogels can be found e.g., in [16,17,18,19]. Non-swelling hydrogels are applied as parts of servo-hydraulic soft robotics [20] or as artificial skins [21]. Hydrogel layer structures were presented e.g., by [21,22]. First works about hydrogel structures that form corrugated sheet patterns were performed by [23]. However, the shell-forming effect is achieved on the micro-level by using buckling mechanisms. The present work uses the different approach of special material combinations and geometry combinations in composite structures in order to achieve this effect on the macro scale.

The modeling and simulation of the hydrogel behavior can be performed on different scales [24,25]. Continuum approaches can treat hydrogels as homogenized materials with swelling behavior [9,26]. If the movement of ions in polyelectrolyte gels has to be resolved, the coupled multifield problem is applied [27,28]. Other approaches are e.g., based on the Theory of Porous Media [29,30,31,32].

**Figure 1:**
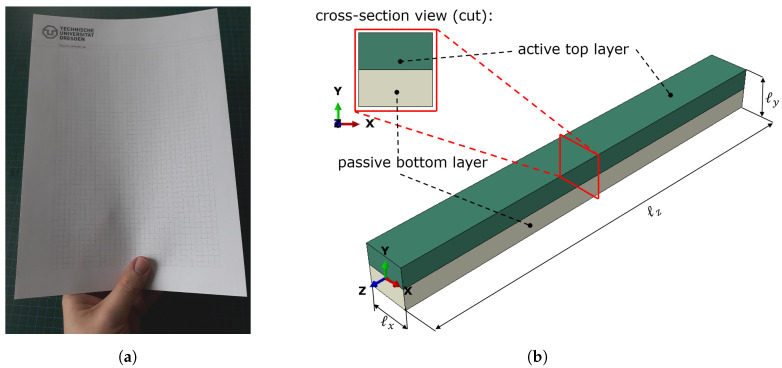
(**a**) Paper held in hand for stabilization. The deformed shape represents an U. In contrast to the flat paper, this U shape has a much higher bending stiffness. In the current work, we show how this effect is applied to (**b**) a beam-like setup by using active materials. Swelling of the active layer leads to a change of the cross-section (*x*-*y*-plane) shape and thus a stabilization. Please note that the beam length axis is the *z*-direction.

The transition from a flat to a curved structure is quite typical: it is intuitively performed by people to stabilize paper sheets when holding them in the hands, see Figure 1a. Curved structures are also prevalent in nature to provide added stability, e.g., for mussel-shells or leaves. The goal of the current work is to mimic this process of shell-forming. We want to highlight the potential of the described structures in micromechanical devices. Especially the capability for drastic stiffness change in structures is worth further investigations.

In the following Section 1.1, we discuss the basics of combining active with passive layers as parts of a composite. The term *stiffness*, which is crucial to the discussion, is explained here, as well. Then, in Section 1.2, we describe the difference between flat and curved structures. In the following, we give the modeling background in Section 2. In the results Section 3, we show how active–passive composites can form shell-like structures. Finally, the conclusion is drawn in Section 4.

### 1.1. Active Hydrogel Composites

In the discussion about combined active–passive structures, the *stiffness* of each component plays a central role. In engineering, stiffness describes a combined value of material and geometry properties of a structure. For homogeneous beam-like structures, like the one in Figure 1b, the curvature $\bar{\kappa }$ of the beam is defined by

(1) $$\begin{array}{c} \bar{\kappa }=-\frac{M_{b}}{K}=-\frac{M_{b}}{E\;I}, \end{array}$$

where *E* is the elastic modulus, *I* the area moment of inertia and $M_{b}$ the bending moment load.

The bending stiffness (here: flexural rigidity) K=EI therefore includes both a material parameter *E* and a geometry parameter *I*. This relation is crucial, since the same bending behavior under identical load can be obtained with different pairings of the material and the geometry parameter. In simple beam cross-section geometries, the area moment of inertia *I* scales with the power of three in height and linearly in width. A slight increase in height is therefore very effective in increasing the total stiffness of a structure without changing the material. This is used e.g., in profiled steel bearings with I-profiles. For solid bulk structures the relationship is much more complex. Simulation studies can give a deeper insight into the behavior of these structures.

Please note that Equation (1) is defined for homogeneous beams. For multi-layer structures involving different materials, the combined behavior can e.g., be modeled using the Classical Laminated Plate Theory [1]. In those cases, a modified *K* can be found for the combined structure. In the current work, we use the same elastic modulus for both, active and passive layer for simplification. Then, Equation (1) can be applied as well.

**Figure 2:**
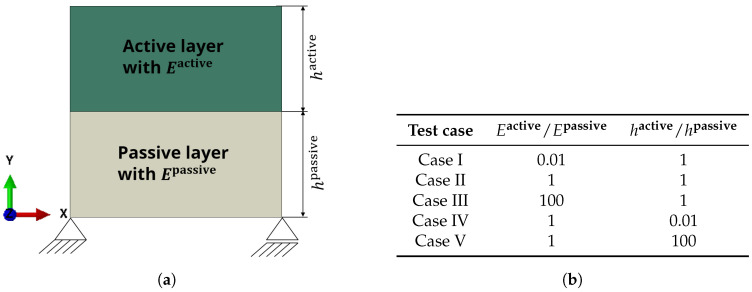
For the depicted setup, the elastic modulus and the height of the active and passive layer is varied to form Case I to Case V. The two-dimensional structure is fixed at the left side with a fixed bearing and at the right side with a floating bearing. (**a**) Base setup of the active–passive pairing. (**b**) Description of the Cases.

We start with preliminary considerations concerning a 2D quadratically shaped structure that consists of two layers, see Figure 2. Here, the structure represents the cross-section of a beam. Please note that the bearings are added to avoid free body movements and rotations when performing exemplary Finite-Element simulations of this setup. In fact, they represent the symmetry condition and the influence of the beam in length-direction.

The upper layer (green) is made of an active material that can perform isotropic expansion, e.g., the hydrogel poly(*N*-isopropyl acrylamide). It is firmly attached to a lower passive layer (gray). We now vary the elastic moduli and the heights of the two layers according to the table in Figure 2b.

It has to be mentioned that the absolute value of the elastic moduli and heights do not play a role in the ensuing overall behavior of the structure. Therefore, only normalized values are depicted that are in relation to the elastic modulus of a known material.

**Figure 3:**
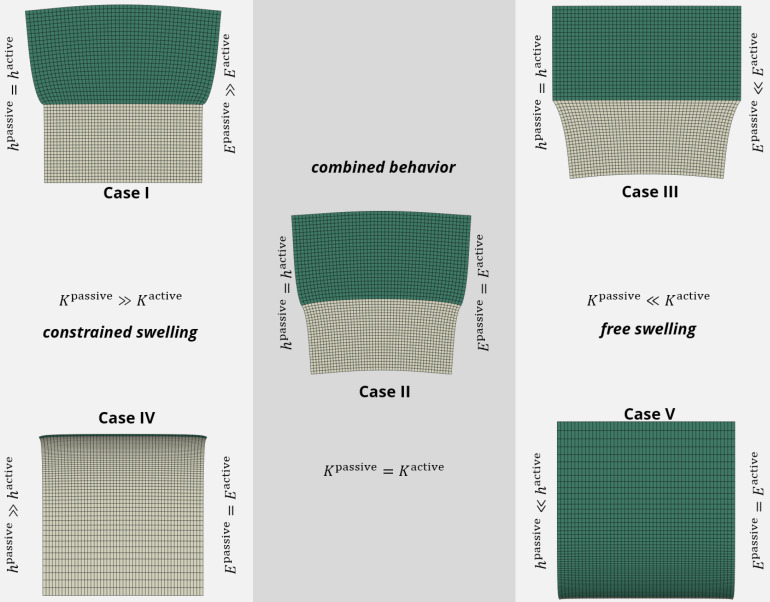
Comparison of different stiffness combinations between the active layer (upper, green) and the passive layer (lower, gray). The fixing is realized according to Figure 2a.

For the test cases given in the table in Figure 2b, the behavior of the structure is depicted in Figure 3. If the hydrogel swelling is isotropic, we can identify three different kinds of resulting behavior of the structure.

**Constrained swelling**: In Cases I and Case IV, the stiffness of the passive layer is much larger than the one of the active layer, i.e., $K^{\mathrm{passive}}\gg K^{\mathrm{active}}$. Therefore, the overall structure is not perceivably influenced by the swelling or deswelling of the active material. Structures of this kind are used technologically in various ways, e.g., for an active hydrogel layer on a glass substrate for cell culture [33,34]. The constrained swelling of this cell cultivation layer does not bend the glass substrate, however, the swelling serves for detaching the cells. Another application of this stiffness combination was presented in our previous work [4]. There, the soft active hydrogel (PNiPAAm) was fixed on a stiff backbone membrane (biaxially oriented polyethylene terepthalate, PET). The constrained swelling leads to an opening and closing of prefabricated pores [4].

**Free swelling**: Case III and Case V feature a very soft passive layer in comparison to the active layer, i.e., $K^{\mathrm{passive}}\ll K^{\mathrm{active}}$. In such structures, the overall behavior is dominated by the free swelling of the hydrogel. For example, a very thin porous graphite electrode on a hydrogel does not hinder the hydrogel in its free swelling. However, it can conduct electricity, which provides an additional function to the structure. Graphite spray can also be used to make hydrogels more opaque to allow the deformation measurement with optical methods [16].

**Combined deformation**: The most interesting case in context of the present research is Case II where the behavior of the overall structure depends on the material pairing, i.e., when $K^{\mathrm{passive}}\approx K^{\mathrm{active}}$. For a bilayer-beam-like structure, only Case II types of combinations will lead to a bending behavior under isotropic swelling of the hydrogel.

If the difference in material elastic moduli is high (e.g., when pairing a hydrogel with a metal), this has to be compensated through the relation of the layer thickness (thin metal foil and thick hydrogel) in order to achieve stiffnesses for active and passive layer in the same order of magnitude. This is used e.g., in sensor setups [2,35]: a very thin silicone membrane deforms under the hydrogel swelling and leads to a sensor output. Even though the silicone has a much higher elastic modulus, the membrane is chosen adequately thin, so that a combined deformation between hydrogel and membrane is observed.

Combinations of isotropic hydrogels with anisotropic passive materials can fall under different categories, depending on the direction. For example, for fiber-reinforced hydrogel composites, the behavior in fiber-direction is dominated by the fibers due to their very high elastic modulus (Case I). In perpendicular direction, the cross-section of the fibers is very small in comparison to the hydrogel matrix (Case V). Therefore, the behavior in this direction is dominated by the free swelling.

From the preliminary considerations, we can conclude that for the stabilization of e.g., flexible displays, Case II is desirable. The actual stiffness (material properties and geometry) of the flexible display heavily depends on the used display technology. However, with the knowledge about stiffness relations, the Case II behavior can be achieved with many of these display technologies.

### 1.2. Passive Shells and Corrugated Sheets

**Figure 4:**
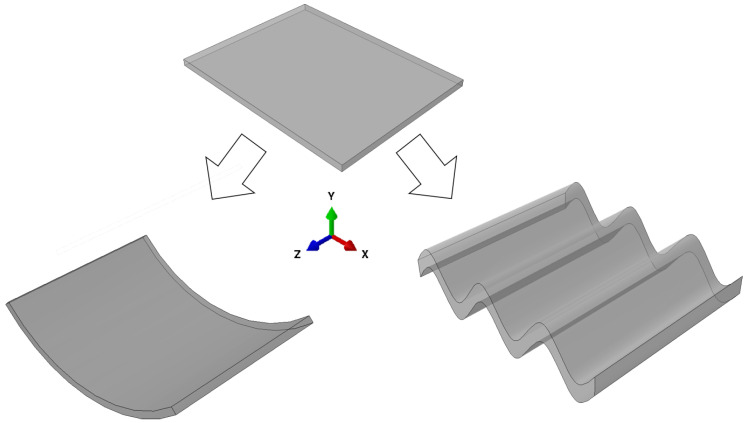
A plate-like structure has a much lower bending stiffness around the *x*-axis than a shell-like structure (**left**) or corrugated sheet structure (**right**) with the same volume.

Shells are solid structures with arbitrary curvature [36]. The theoretical background of the modeling of composite shells is quite extensive [36,37]. Especially the integration of the active behavior and the combination of materials are an ongoing research field. Since the goal of the current work is to discuss the concept of shell-forming hydrogel composites, we will not go deeply into the modeling considerations. Instead, we focus on the bending behavior of Bernoulli beams described in Equation (1). This simple case provides basic insights about the stiffness change of structures under bending. Future quantitative works have to include advanced modeling techniques to adequately represent the shell-like behavior. In Figure 4, a plate and the same structure in its curved U shape and corrugated sheet shape are depicted.

The basic concept of corrugated sheets is to use the geometric stiffening effect of the curvature also on larger surfaces, see Figure 4. For example, corrugated steel can provide a cheap and stable roofing. It is easily produced by inducing local plastic deformations e.g., through roll forming. In the current work, we present a concept of reversibly inducing local swelling and deswelling for the formation of corrugated sheets. This is done using an active–passive composite made (i) of a hydrogel and (ii) of a passive material. According to Case II (see Section 1.1), the stiffness of both components must be in the same order of magnitude to allow deformation of the combined structure.

Please note that the current effect is not due to buckling as it is described e.g., in [23,38]. However, in the current work, no instability is observed since the active–passive composite bends to reduce the occurring stresses inside the material without reaching bifurcation points.

## 2. Modeling of Composite Shells Using the Temperature-Expansion-Model

**Figure 5:**
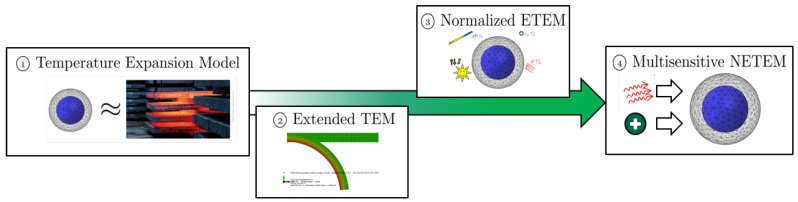
Development of the hydrogel model:①: Temperature-Expansion-Model (TEM) [4] →②: Extended TEM (ETEM) [39] →③: Normalized ETEM (NETEM) [1,3,9,40,41] →④: Multisensitive NETEM (M-NETEM) [42].

In our previous works, we proposed a model for the isotropic swelling of hydrogels. It is called Temperature-Expansion-Model (TEM) and it is based on the analogy of isotopic swelling and thermal expansion. In our previous work [4,39], the model was defined for linear elasticity and geometry. We showed that the model (i) is easy to implement and (ii) shows excellent agreement with experimental data. In the next step, we extended the model with respect to nonlinear geometry [40]. The extended TEM (ETEM) allows the simulation of hydrogel structures with large deformations. This was followed by another extension which we called the Normalized ETEM (NETEM). With the normalization procedure, arbitrary stimuli that lead to isotropic swelling can be incorporated, not only temperature [9,40,41]. The newest addition is the incorporation of multisensitive behavior, the M-NETEM [42], see Figure 5.

The NETEM is applied in course of this work in order to obtain insights about the shell-forming of hydrogel composites. The following set of equations describes the continuum mechanical background of thermal expansion.

(2) $$\begin{array}{ccc} \mathrm{Balance}\;\mathrm{laws}: & \; & \sigma_{kl,k}+f_{l}=0\;\sigma_{kl}=\sigma_{lk} \end{array}$$

(3) $$\begin{array}{ccc} \mathrm{Kinematics}: & \; & \varepsilon_{kl}^{H}=\frac{1}{2}\ln\left(B_{kl}\right) \end{array}$$

(4) $$\begin{array}{ccc} \mathrm{Constitutive}\;\mathrm{relation}: & \; & \sigma_{kl}=E_{klmn}\left(\varepsilon_{mn}^{H}-\underset{\mathrm{expansion}\;\mathrm{strain}}{\underset{︸}{\beta \left(F^{\mathrm{Stimulus}}\right)\;\delta_{mn}\;\Delta F^{\mathrm{Stimulus}}}}\right), \end{array}$$

where, $\sigma_{kl}$ denotes the true stress, $f_{l}$ the volume loads and $\varepsilon_{kl}^{H}$ the logarithmic Hencky strain with $B_{kl}=F_{kM}\;F_{lM}$ being the left Cauchy-Green deformation tensor based on the deformation gradient $F_{kl}$. In the Saint–Venant–Kirchhoff-like linear elastic material behavior, the additional term is the expansion strain. It is composed of the isotropic stimulus expansion coefficient $\beta (F^{\mathrm{Stimulus}})$ and the difference in stimulus ratio $\Delta F^{\mathrm{Stimulus}}=\left(F^{\mathrm{Stimulus}}-1\right)$ which denotes the stimulus influence. The Kronecker-delta is denoted by $\delta_{mn}$. The indices k,l,m,n∈[x,y,z] denote the respective tensor base $e_{x},e_{y},e_{z}$ and the Einstein summation convention holds.

Please note that in the current work, we do not focus on specific material behavior. This can be found in our previous works, see e.g., [9,42]. Instead, we use exemplary material data for design purpose. In order to allow the comparison to experimental data, the expansion strain is chosen in the same order of magnitude (≈30%) as the swelling of PNiPAAm in our previous works [4]. Hence, in the following simulation studies, β=0.1 was chosen. A stimulus ratio change of $\Delta F^{\mathrm{Stimulus}}=1$ (which is modeled by a pseudo-temperature change of 1K) therefore leads to 10% expansion strain.

Based on the Normalized Extended Temperature-Expansion-Model, we performed simulations using the commercial Finite-Element tool Abaqus. The corresponding settings to reproduce this model are nonlinear kinematics with linear elasticity and thermal expansion. No additional implementation steps were needed, since thermo-elasticity is a well-known problem in both academic and industrial problems.

## 3. Simulation Results for Shell-Forming Hydrogel Composites and Active Corrugated Sheets

In the current section, simulation results for the shell-formation are given. At first, we concentrate on simple two-layer structures that form an U shape under swelling, see Section 3.1. Then, the concept is extended to structures that form basic corrugated sheet setups, see Section 3.2.

Please note that in the following simulation studies, we focus on the swelling of hydrogels inside the structures, i.e., a positive volume expansion. In addition, the materials’ elastic moduli are chosen equal $E^{\mathrm{active}}=E^{\mathrm{passive}}$, according to the discussion from Section 1.1. Therefore, in the current work, all stiffness changes result from geometry changes only.

### 3.1. Simple Curved Beam-Like (U-forming) Structure

The investigated structure is a beam-like structure with width of $\ell_{x}=100\mathrm{mm}$ in *x*-direction, height of $\ell_{y}=10\mathrm{mm}$ in *y*-direction and length of $\ell_{z}=1000\mathrm{mm}$ in *z*-direction.

**Figure 6:**
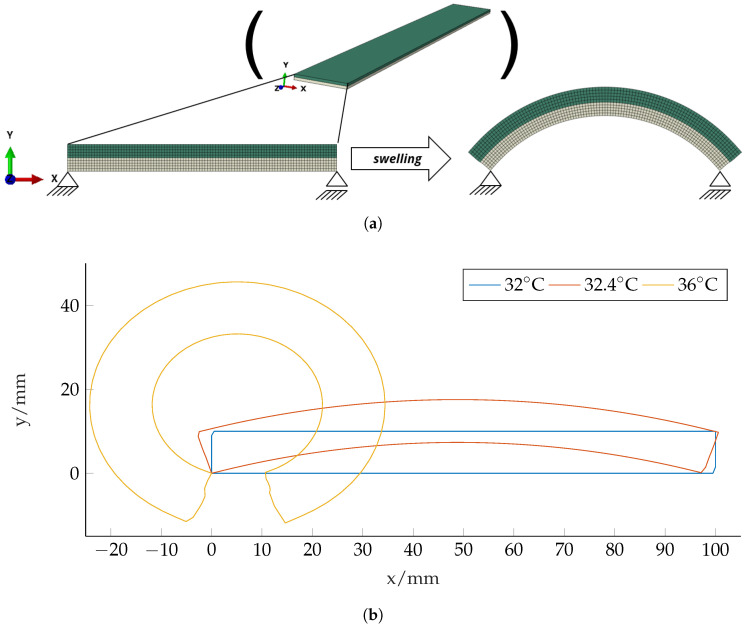
Basic concept and 2D simulation results for the cross-section of the U-forming beam made of two layers. (**a**) Two-dimensional swelling of an active–passive composite structure. The structure represents the cross-section of a beam according to Figure 1b, the beam-length direction is out of plane (*z*-direction). In this Finite-Element simulation result, we observe the wanted effect from Figure 1a: under swelling, the cross-section forms an inverted U which leads to an increased bending stiffness. (**b**) Beam cross-section at different temperature states and according two-layer structure.

Two-dimensional preliminary simulations of the beam cross-section show the desired effect, see Figure 6a. The rectangular shaped cross-section in the *x*-*y*-plane bends around the *z*-axis due to swelling and active–passive interaction similar to Case II in Figure 3. With increasing swelling, the rectangular shaped cross-section transforms into an inverted U-profile and finally curls up to build an O-shaped hollow profile, see Figure 6b. The results were gained by Abaqus simulations according to Section 2 with 1000 quadratic quadrilateral elements of type CPS8R.

**Figure 7:**
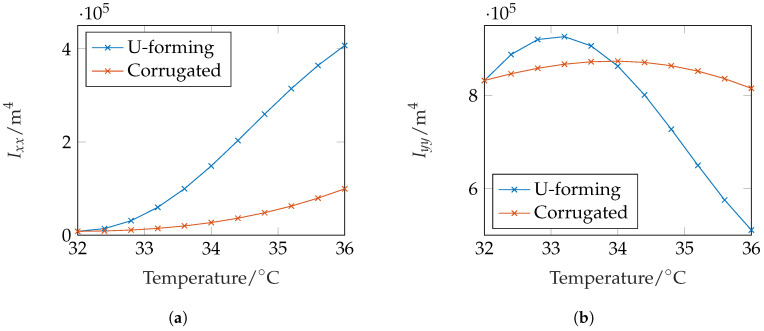
The swelling of the simple two-layer structure according to Figure 6a (U-forming) and the corrugated sheet structure (presented in Section 3.2) lead to a change in *I_xx_* and *I_yy_*. (**a**) Moment of inertia for bending around the x-axis. (**b**) Moment of inertia for bending around the y-axis.

In order to quantify the stiffness change between the configurations, we calculate the moment of inertia related to the coordinate system in the center of mass

(5) $$\begin{array}{c} I_{xx}=\int \int y^{2}\;dydx \end{array}$$

of the initial and the deformed structures. In Figure 7a the stiffness-change over the temperature is depicted. Please note that for deformed states, the beam neutral axis shifts due to the geometry change. We therefore wrote a Matlab script which calculated $I_{\bar{x}\bar{x}}$ in an outer $\bar{x}$-$\bar{y}$ coordinate system. Then, the center of mass of the cross-section is calculated and Steiner’s theorem (parallel axes theorem) is applied to transform it to the correct axis. For the sake of completeness, we also calculate the geometry influence (in the moment of inertia) for bending around the y-axis

(6) $$\begin{array}{c} I_{yy}=\int \int x^{2}\;dxdy. \end{array}$$

The results of the moment of inertia $I_{yy}$ are depicted in Figure 7. The deviatory moment of inertia which leads to skew bending in unsymmetrical profiles is calculated by

(7) $$\begin{array}{c} I_{xy}=-\int \int x\;y\;dxdy. \end{array}$$

For the U-forming beam cross-section, we obtain an increase of $I_{xx}$ and subsequently a stiffness-change to a maximum of $I_{xx,\max}/I_{xx,\min}\approx 49$. The moment of inertia around the *y*-axis first increases until ϑ≈33.2°C and then decreases again. This is due to the fact that the cross-section (i) elongates in *x*-direction and (ii) bends around the *z*-axis and therefore contracts in its *x*-dimension. For ϑ>33.2°C, the latter effect begins to dominate and $I_{yy}$ decreases reaching a minimum of $I_{yy,\min}/I_{yy,\max}\approx 0.55$. The investigations at the 2D cross-section show that a huge change in $I_{xx}$ can be achieved by forming a simple curved U structure.

**Figure 8:**
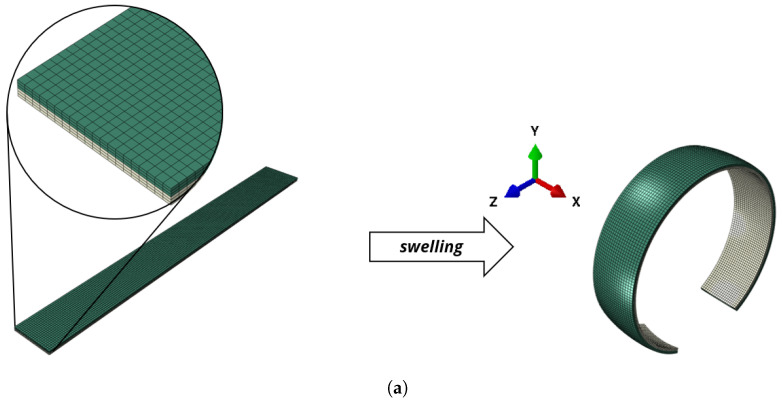

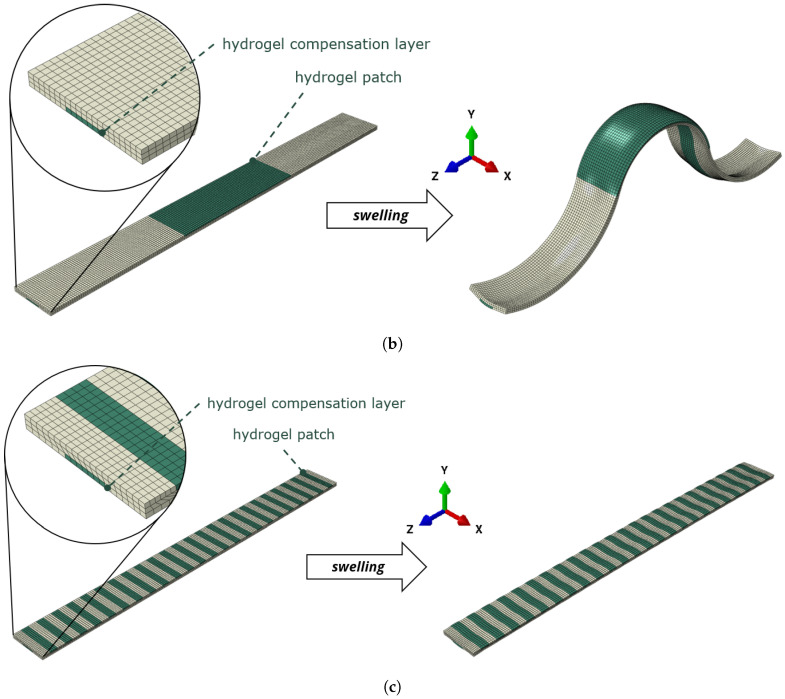
Two-dimensional swelling active–passive composite structure showing the goal for the cross-section of the plate. For all structures, the width in *x*-direction is 100mm, the height in *y*-direction is 10mm and length in *z*-direction is 1000mm. The dimensions of the hydrogel-patches vary according to Table 1. Please note that due to different volumes of active material, the temperatures for the swollen states also vary. (**a**) Two-layer structure with active top layer and passive bottom layer. The temperature of the deformed state is ϑ=32.1°C. The results were obtained with Abaqus according to Section 2 with 24000 linear hexahedral elements of type C3D8R. (**b**) In addition to the compensation strip on the bottom side, one patch is inserted. The temperature of the swollen state is ϑ=33°C. The results were obtained with Abaqus according to Section 2 with 14652 quadratic hexahedral elements of type C3D20R. (**c**) In addition to the compensation strip on the bottom side, 24 patches are inserted. The temperature of the swollen state is ϑ=33°C. The results were obtained with Abaqus according to Section 2 with 11880 quadratic hexahedral elements of type C3D20R.

However, this effect cannot directly be transferred to 3D structures. Due to the isotropic swelling of the hydrogel layer, an additional bending around the *x*-axis is obtained. This bending far exceeds the curvature gain (U-forming) due to bending around the *z*-axis, see Figure 8a. In the current application case for e.g., a flexible display, the additional bending is not desirable.

Therefore, a compensation layer has to be added which counteracts this bending. In the current work, we included a compensation layer on the bottom side with the dimensions 33.3mm×3.33mm×1000mm, see Figure 8b. The compensation layer spans the whole length of the beam and therefore exercises a high bending influence around the *x*-axis. However, the bending influence around the *z*-axis (which counteracts the U-forming) is small since it only spans over a third of the beam width.

The hydrogel patches on the top side lead to a bending around the *z*-axis according to Figure 6a and therefore the resulting U-forming. In contrast to the compensation layer, their influence towards U-forming is much higher. The dimensions of the patches are given in Table 1.

The setup with a compensation layer and one patch leads to a wave form under swelling, see Figure 8b. This effect can be mitigated by using various patches of incorporated hydrogel and an adequate hydrogel compensation layer on the bottom side, see Figure 8c. This structure first starts to bend in the same way as the one in Figure 8a and returns back to a flat wave form during the swelling process.

In these investigations, it can be seen that in order to achieve a system without bending in beam length direction (around the *x*-axis), the longitudinal component (in *z*-direction) of the swelling has to be compensated. Additional studies have shown that this cannot be obtained by simply including the same volume of hydrogel at both sides of the beam neutral axis; instead the hydrogel compensation layer (lower side) has to be smaller. We assume that this effect is due to the influence of the geometrically nonlinear model.

The studies with a simple beam setup for shell-forming structures have shown that a two-layer structure is not suitable for achieving the envisioned stiffening effect for flexible displays. However, the problem of bending around the *x*-axis can be avoided by using anisotropic materials that show a much higher elastic modulus in longitudinal direction than in transverse and thickness direction, i.e., $E_{z}\gg E_{x},E_{y}$. Then, a case similar to Case I in Figure 3 would be obtained in the *x*-*y*-plane and no unwanted bending would occur. However, this would also decrease the change of bending stiffness in this direction because the high $E_{z}$ has a much stronger influence on the bending stiffness than the shell-formation. Therefore, in further studies, we will focus on new material and geometry combinations to achieve an adequate U-forming.

### 3.2. Active Corrugated Sheets

**Figure 9:**
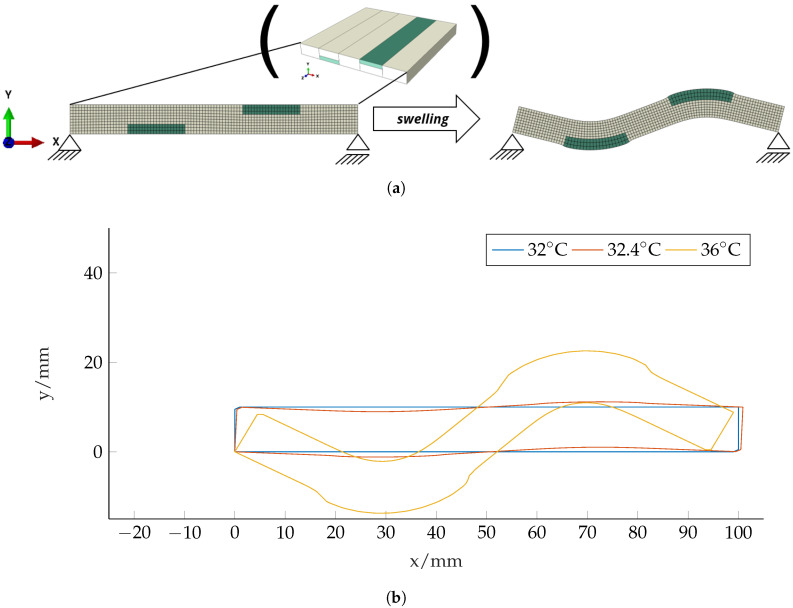
Basic concept and 2D simulation results for the cross-section of a beam made to form a corrugated sheet. (**a**) Two-dimensional swelling of an active–passive composite structure that represents the cross-section of a plate according to Figure 4. Under swelling, the cross-section forms a corrugated sheet-like structure. The two hydrogel patches have the dimensions 20mm×3.33mm. Please note that this is a different setup in comparison to the U-forming beam structure. (**b**) Beam ross-section at different temperature states and according two-layer structure.

In active corrugated sheets, multiple layers of hydrogel are included on both sides of the plate. Therefore, the bending around the *x*-axis due to swelling is compensated and only the shell-formation remains. The basic concept is shown in a two-dimensional simulation, see Figure 9a. Here, the two included hydrogel patches lead to a S-forming of the structure. The results were gained by Abaqus simulations according to Section 2 with 1000 quadratic quadrilateral elements of type CPS8R.

In Figure 7a, the effect of change of the moment of inertia $I_{xx}$ is depicted. In comparison to the U-forming, the change is much smaller with $I_{xx,\max}/I_{xx,\min}\approx 12$. However, this is only due to the much smaller volume of active material in comparison to the volume of passive material. The geometry influence of $I_{yy}$ to the bending stiffness around the *y*-axis remains nearly the same with a maximal change of $I_{yy,\min}/I_{yy,\max}\approx 0.93$. Due to the non mirror symmetric setup, the deviatory moment of inertia is not zero, i.e., $I_{xy}\neq 0$. Therefore, we expect skew bending of a beam-like structure with this cross-section.

**Figure 10:**
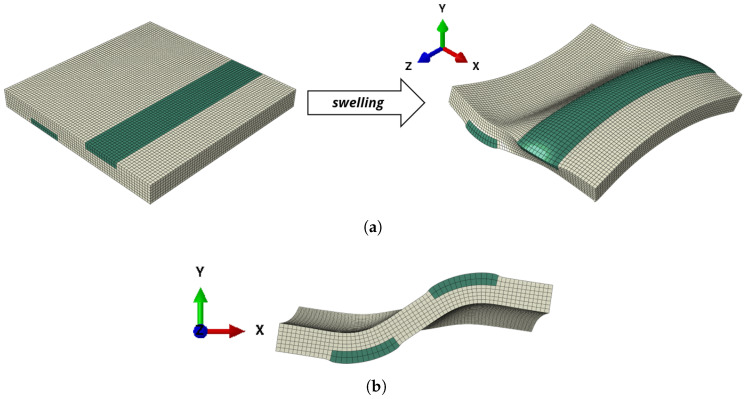
Rectangular plate-like structure with width in x-direction $\ell_{x}=100\mathrm{mm}$, height in *y*-direction $\ell_{x}=10\mathrm{mm}$ and length in *z*-direction $\ell_{x}=100\mathrm{mm}$. ue to the included hydrogel patches, an S-form according to Figure 9 evolves under swelling. (**a**) Structure that forms a corrugated shell. The deformed state is at ϑ=34°C. The dimensions of the hydrogel patches are equal to the ones in Figure 9. The results were obtained with Abaqus according to Section 2 with 30485 quadratic hexahedral elements of type C3D20R. (**b**) Cut through the deformed state at the middle $z_{\mathrm{cut}}=50\mathrm{mm}$.

In the three-dimensional setup, we see that due to the opposed layers, no bending around the *x*-axis is observed, see Figure 10a. However, we observe warping (bitorsion) at the front and back side of the composite plate. Therefore, depending on the fixing, additional stresses in longitudinal direction can occur. In Figure 10, a cut through the deformed structure is shown. Since it is in the middle of the structure, due to symmetry, the warping displacement cancels out at this cut. An S-shape as shown in Figure 9 can be observed.

These preliminary studies about the corrugated sheet forming structure are more promising than the U-forming structures above. There is no need to compensate an unwanted bending around the *x*-axis. Future studies can now focus on the optimization of corrugated sheet forming hydrogel composites. This includes size and positioning of the patches in relation to the passive material.

## 4. Conclusions and Outlook

In the present concept study, a combined active–passive structure has been investigated. It was shown that the swelling capacities in active–passive structures can be used in a different way that usual muscle-like linear actuators or simple benders. Instead, in the discussed active–passive structures, the hydrogel itself is not load-bearing, but helps to form a shell-structure. This indirect actuation leads to a drastic increase in bending stiffness due to geometry change.

Numerical simulations using the Temperature-Expansion-Model allow the quantification of this stiffness change. Based on preliminary considerations about material pairing, we analyzed structures with (i) U-forming and (ii) S-forming cross-sections. (i) Simple two-layer composites form an U shape when the active material swells. However, an adequate 3D setup is difficult to achieve because an unwanted bending effect far outruns the stiffening effect. A compensation of this unwanted bending also mitigates the shell-forming and therefore decreases its stiffening effect. (ii) With a distribution of the active material that leads to S-forming cross-sections, this effect does not appear. However, warping of the cross-section appears which may lead to additional stresses in the material.

Future works in this direction will focus on the experimental realization of such systems. In addition, the Temperature-Expansion-Model can be included in advanced composite shell theories to allow arbitrarily curved surfaces.

The developed concept of active–passive composites can be directly applied to rollable/flexible displays: In its unactuated state, the structure has a very low bending stiffness and can be rolled up. Under actuation, a corrugated sheet setup is formed and the display is then stiff. Therefore, the integration of stiffening active material layers could replace hard casings in devices with flexible displays.

## Figures and Tables

**Table 1:** Test cases and geometry for the U-forming beam structure. The geometry is given as width × height × length, i.e., $\ell_{x}\times \ell_{y}\times \ell_{z}$.

Case	Number of Patches	Patch Geometry [mm]	Figure
Ubeam0	0	100×50×1000	Figure 8a
Ubeam1	1	100×33.3×333.3	Figure 8b
UbeamN	24	100×33.3×20	Figure 8c
